# Strong Coupling of Coherent Phonons to Excitons in Semiconducting Monolayer MoTe_2_

**DOI:** 10.1021/acs.nanolett.3c01936

**Published:** 2023-09-26

**Authors:** Charles J. Sayers, Armando Genco, Chiara Trovatello, Stefano Dal Conte, Vladislav O. Khaustov, Jorge Cervantes-Villanueva, Davide Sangalli, Alejandro Molina-Sanchez, Camilla Coletti, Christoph Gadermaier, Giulio Cerullo

**Affiliations:** † Dipartimento di Fisica, Politecnico di Milano, 20133 Milano, Italy; ‡ Center for Nanotechnology Innovation @ NEST, Istituto Italiano di Tecnologia, 56127 Pisa, Italy; ¶ Institute of Materials Science (ICMUV), University of Valencia, Catedrático Beltrán 2, E-46980 Valencia, Spain; § Division of Ultrafast Processes in Materials (FLASHit), Istituto di Struttura della Materia-CNR (ISM-CNR), Area della Ricerca di Roma 1, 00016 Monterotondo, Scalo, Italy; ∥ Department of Mechanical Engineering, Columbia University, New York, New York 10027, United States; ⊥ Scuola Normale Superiore, Piazza San Silvestro 12, 56127 Pisa, Italy; # Graphene Labs, Istituto Italiano di Tecnologia, 16163 Genova, Italy

**Keywords:** Coherent phonons, excitons, ultrafast spectroscopy, transition metal dichalcogenides, two-dimensional materials, monolayer, MoTe_2_

## Abstract

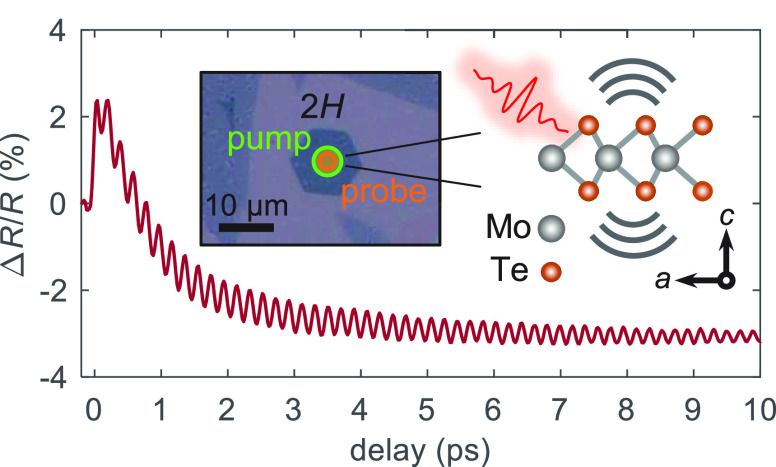

The coupling of the electron system to lattice vibrations and their time-dependent control and detection provide unique insight into the nonequilibrium physics of semiconductors. Here, we investigate the ultrafast transient response of semiconducting monolayer 2*H*-MoTe_2_ encapsulated with *h*BN using broadband optical pump–probe microscopy. The sub-40 fs pump pulse triggers extremely intense and long-lived coherent oscillations in the spectral region of the A′ and B′ exciton resonances, up to ∼20% of the maximum transient signal, due to the displacive excitation of the out-of-plane *A*_1g_ phonon. Ab initio calculations reveal a dramatic rearrangement of the optical absorption of monolayer MoTe_2_ induced by an out-of-plane stretching and compression of the crystal lattice, consistent with an *A*_1g_ -type oscillation. Our results highlight the extreme sensitivity of the optical properties of monolayer TMDs to small structural modifications and their manipulation with light.

Electron–phonon coupling is crucially important to many phenomena in condensed matter, such as carrier scattering in transport,^1−3^ the relaxation of photoinduced nonequilibrium quasiparticle populations,^4−10^ and electronic order emerging at low temperatures.^11−14^ Photoexcitation of coherent phonons (CPs) using ultrashort light pulses enables fundamental insight into electron–phonon interactions via their excitation and detection mechanisms,^15−17^ which has led to discoveries such as elucidating the role of vibrational coherence in the primary event of vision,^18^ detecting coherent Bloch oscillations in coupled semiconductor quantum wells,^19^ and demonstrating THz radiation emission due to the macroscopic polarization originating from CPs.^20^ There is potential for applications in sensors, actuators, and transducers^21−24^ operating at frequencies up to several THz.^25^ In semiconductors with large exciton binding energies, photoexcitation of CPs has provided vital information about exciton–phonon coupling.^26−29^

Two-dimensional semiconductors such as monolayer transition-metal dichalcogenides (TMDs) combine strong light-matter interaction and multifaceted exciton and valley physics with a great potential for applications in energy harvesting and information processing.^30−35^ An important tool in the investigation of exciton-coherent phonon coupling in these materials is femtosecond transient absorption (TA) spectroscopy. Here, an initial ultrashort pump pulse photoexcites the sample, while a second delayed probe pulse is used to measure the transient change in the optical response. This allows for tracking the temporal evolution of the nonequilibrium quasiparticle populations, which manifests as the characteristic decay time of the TA signal. In WSe_2_, it has previously been shown that coherent oscillations of the out-of-plane A_1g_ phonon mode introduce a small modulation (∼10^–3^ of the transient signal) on the electronic relaxation at the optical bandgap.^36^ Similarly, in monolayer MoS_2_, the oscillatory modulation of the TA signal has also been ascribed to CPs belonging to the *A*_1g_ mode, which exhibit a relatively small amplitude across the spectral region of the A and B exciton resonances,^36^ but a significant modulation of ∼2% of the maximum signal around the C exciton.^28^

MoTe_2_ is a TMD with two well-known thermodynamically stable polymorphs with distinct electronic properties. Its semimetallic phase,^37,38^ (1*T*′ above and *T*_*d*_ below 240 K) exhibits large magnetoresistance,^39^ ferroelectricity,^40^ and superconductivity.^41^ The semiconducting 2*H* phase, on the other hand, has an indirect bandgap in the bulk which becomes direct (∼1.1 eV) toward the monolayer limit,^42,43^ thus expanding the potential functionality of TMDs into the near-infrared (NIR). Furthermore, a high carrier mobility,^44^ strong spin–orbit coupling,^45^ valley selectivity,^46^ and ambipolar transistor behavior,^47^ make it a promising candidate for NIR optoelectronics, photovoltaics, and unconventional information encoding such as spintronics or valleytronics. However, since its lower chemical stability has been overcome only recently by encapsulation using few-layer *h*BN,^48^ MoTe_2_ is significantly less studied than its sulfur and selenium analogues. Previous TA experiments on 2*H*-MoTe_2_ with various probe energies, ranging from 1.0 eV to 2.6 eV, have elucidated the dynamics of several excitonic transitions, but without detecting any CP signature thus far.^49−51^ Optical pump-core level (XUV) probe spectroscopy, on the other hand, has revealed a strong oscillatory signal contribution dominated by the out-of-plane *A*_1g_ mode with a smaller *E*_1*g*_ component,^52^ where the greatest oscillation amplitude was observed for transitions from the Te-4*d*_5/2_ levels to the conduction band.

Here, we employ broadband TA microscopy to study the coupling of the out-of-plane *A*_1g_ vibrational mode in semiconducting monolayer 2*H*-MoTe_2_ to several excitonic resonances. Our work is supported by ab initio simulations, which combine density functional and perturbation theory (DFT/DFPT) with many-body perturbation theory (GW+BSE). We find an exceptionally strong and long-lived oscillatory signal contribution, which is rarely observed in semiconducting TMDs. Our broadband probe combined with an excellent temporal resolution of ≤40 fs reveals the spectral dependence of the amplitude and phase of CPs with exceptional clarity. Our simulations confirm a strong modulation of the electronic band structure and, consequently, the absorption spectrum by out-of-plane atomic motion, allowing the theoretical prediction of the spectral profile of the oscillation amplitude in excellent agreement with the experimental observations. Our results demonstrate how the optical properties of monolayer MoTe_2_ in the visible and NIR range are highly susceptible to manipulation via small structural modifications and how these can be controlled optically using ultrashort light pulses.

Samples of MoTe_2_ were synthesized by chemical vapor deposition on Si/SiO_2_ and encapsulated with few-layer *h*BN according to the methods in ref (48). The procedure yields flakes of both polymorphs, which are easily distinguished by their shape; elongated for 1*T*′, or hexagonal for 2*H*. Since the 2*H* flakes have a lateral size of only a few micrometers, we employed a broadband optical pump–probe microscope^53^ whereby pump and probe are focused onto the sample using an objective lens, as illustrated in Figure 1a, providing a spatial resolution of ∼3 μm. The Raman spectrum measured on the same flake, shown in Figure 1b, confirms the semiconducting 2*H* polymorph, with the most prominent peak at 235 cm^−1^ originating from the in-plane *E*_2g_ phonon. A further peak, seen here at 170 cm^−1^, is associated with the out-of-plane *A*_1g_ phonon and has been shown in previous studies to be clearly visible for excitation at 633 nm (1.96 eV), but much weaker for excitation at 532 nm (2.33 eV).^42^ The absence of modes at 120 and 290 cm^−1^ confirms the flake to be monolayer.^42,54^ The calculated absorption spectrum of monolayer 2*H*-MoTe_2_, shown in Figure 1c, exhibits a series of excitonic resonances, whose energies and relative intensities match closely with previously measured spectra.^42,55^

**Figure 1:**
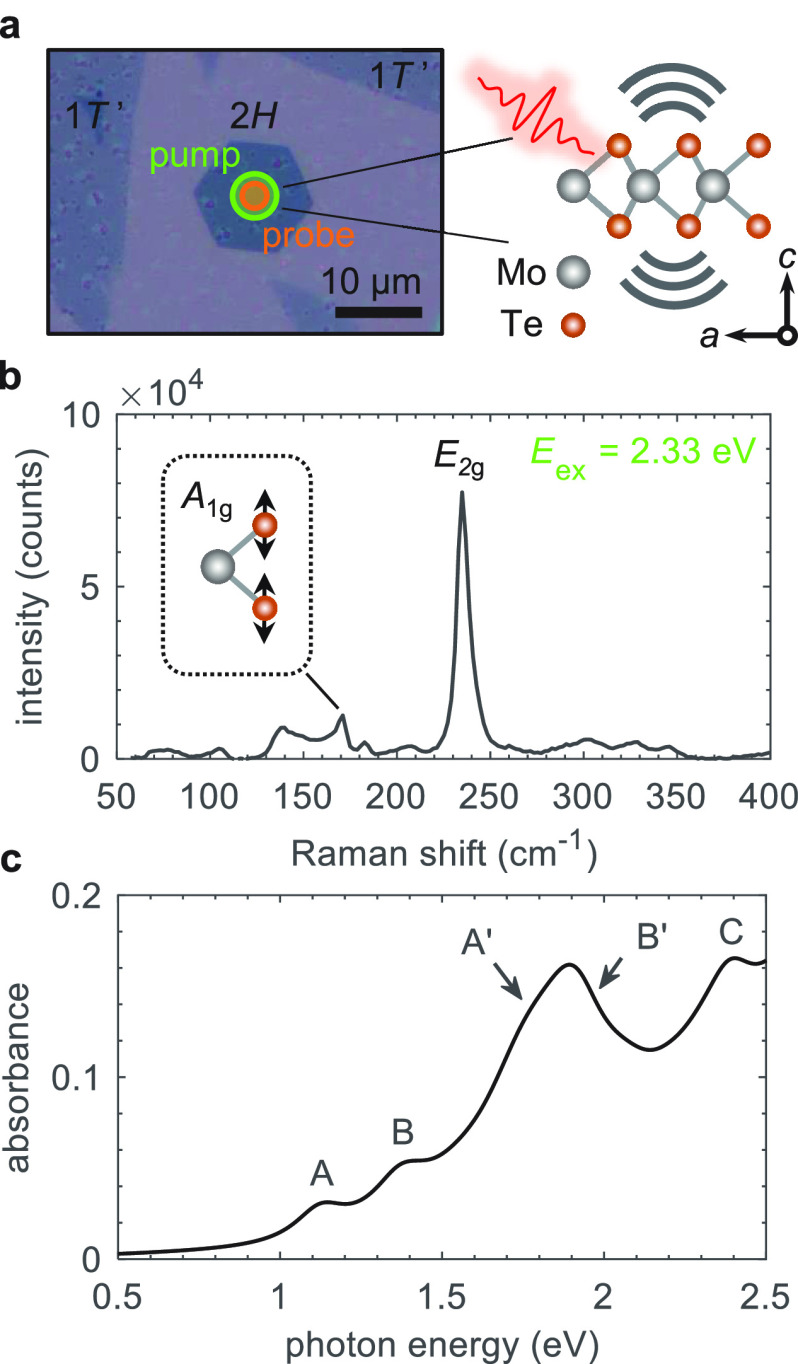
Optical properties of monolayer 2*H*-MoTe_2_. (a) Microscope image of *h*BN encapsulated monolayer MoTe_2_ samples on Si/SiO_2_ (left). Optical pump–probe microscopy experiments were performed on the 2*H* region with pump (∼5 μm) and probe (∼3 μm) beam diameters, as illustrated. The optical pulse launches an intense out-of-plane (*c*-axis) vibration of the lattice (right). (b) Raman spectrum of the MoTe_2_ sample measured with 532 nm (∼2.33 eV) excitation. The out-of-plane vibration with *A*_1g_ symmetry is highlighted. (c) Optical absorption spectrum for the equilibrium structure from ab initio calculations. Optical transitions are labeled according to the convention of ref (55).

We now investigate the transient dynamics of monolayer MoTe_2_ at *T* = 10 K after photoexcitation with a pump centered at ∼2.36 eV. We measure the differential reflectance, Δ*R*/*R* with a broadband probe in the range ∼1.7–2.6 eV at variable delay after excitation. The pump and probe beams are focused and spatially overlapped on the sample, as indicated in Figure 1a. The probe is detected in backscattering geometry after interaction with the *h*BN-MoTe_2_–Si/SiO_2_ sample stack. The dominant effect of photoexcitation is a change in the absorption spectrum of the MoTe_2_ layer, and, hence, we assume the measured differential reflectance, Δ*R*/*R* to be proportional to Δ*A*, i.e., the change in absorbance of MoTe_2_. Further details are provided in the Methods section of the Supporting Information.

The transient Δ*R*/*R* spectra, shown over the first 5 ps in Figure 2a, exhibit two positive bands of increased reflectivity upon photoexcitation and two negative bands of decreased reflectivity. The positive Δ*R*/*R* signal is ascribed to the photobleaching (PB) of the excitonic transitions. The PB peaks from 1.75 eV to 2.2 eV and above 2.4 eV match the positions of the A′, B′, and C excitonic resonances according to ref (42) (see also Figure 2b), suggesting a reduced absorption due to Pauli blocking. The negative Δ*R*/*R* signal instead originates from exciton energy renormalization, which causes a shift of the transition and induces a change of sign in the pump–probe signal, from 2.2 eV to 2.4 eV and below 1.75 eV.^56,57^ Immediately after photoexcitation, an increase of the electronic temperature broadens the exciton line shape, which subsequently narrows at longer delay times. The extracted exciton dynamics are shown in Figure 2c. After a nearly instantaneous rise time, i.e., limited by the temporal resolution of the experiment, the PB signal related to the A′ and B′ resonances decays with time constants of the order τ_fast_ = 0.7–0.8 ps and τ_slow_ = 6.0–8.0 ps (see Figure S1 in the Supporting Information), without further significant changes of spectral shape.

**Figure 2:**
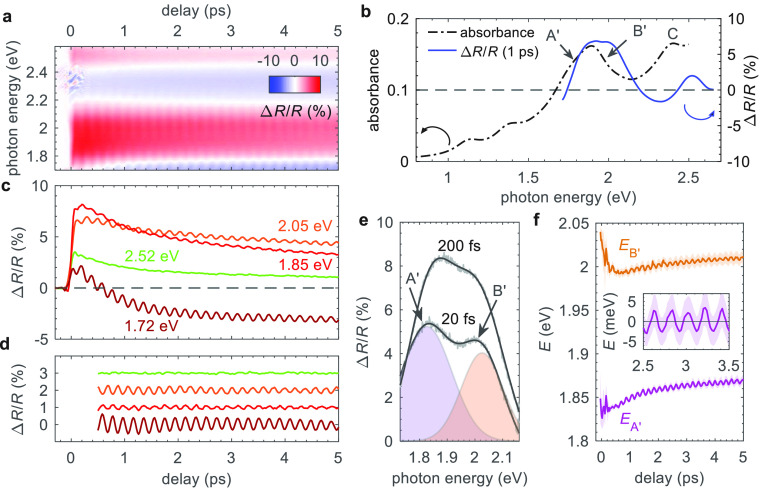
Transient optical response of monolayer 2*H*-MoTe_2_. (a) Broadband differential reflectance (Δ*R*/*R*) maps following excitation with a pump photon energy of 2.36 eV and a fluence of 500 μJ cm^–2^. The sample temperature was 10 K. (b) Transient Δ*R*/*R* spectrum (right axis) at 1 ps delay compared to the calculated optical absorbance of the equilibrium structure (left axis). (c) Dynamics extracted at various probe photon energies, as indicated. (d) Isolated coherent component of the Δ*R*/*R* signal after subtraction of a biexponential fit to the incoherent dynamics in panel (c). Data are offset for clarity. (e) Δ*R*/*R* spectra at early times (20 and 200 fs) showing two positive peaks related to the A′ and B′ transitions. The bold solid lines are fits to the data using a multiple Gaussian procedure, where the shaded areas show the two individual components for the A′ (purple) and B′ (orange) peaks. (f) Temporal evolution of the peak center energies, *E*_A′_ and *E*_B′_ obtained from the fitting in panel (e). The inset shows the energy modulation of the *E*_A′_ peak after subtraction of a biexponential. The shaded areas are the associated fitting errors.

We now turn our attention to the oscillations, clearly visible in the spectral region from 1.7 eV to 2.3 eV (see Figures 2a and 2c), which we assign to photoexcited CPs.^28^ Such oscillations have not been observed in previous work on MoTe_2_ for a probe in the visible range.^49−51^ Here, however, we find an exceptionally strong coherent (oscillatory) component with a magnitude of up to 20% of the maximum Δ*R*/*R* signal, which is an order of magnitude greater than the CP amplitude (2%) found in monolayer MoS_2_.^28^ The isolated oscillatory component, shown in Figure 2d, obtained by subtracting the incoherent (nonoscillatory) signal contribution with a biexponential decay, can be fitted with a single damped cosine term (see Figure S2 in the Supporting Information). The CP mode, measured at 10 K, has a period of 194 fs, which corresponds to a frequency of 5.15 THz (∼172 cm^−1^), and an energy of 21 meV. The *A*_1g_ Raman mode frequency in Figure 1b, measured instead at 295 K, is 5.10 THz (∼170 cm^−1^), consistent with our observations of the CP mode at 295 K (see Figure S3 in the Supporting Information) and the expected temperature-induced mode softening. Therefore, based on this agreement, and previous observations in pump–probe studies of TMDs,^14,28^ we assign the CP mode to the out-of-plane *A*_1g_ vibration. The CP component lasts for tens of picoseconds and exhibits a damping time of τ_damp_ = (6.25 ± 0.25) ps, suggesting that vibrational dephasing is weak. Interestingly, we find that the phonon lifetime is almost independent of temperature between 10 K and 295 K (see Figure S3), which has also been noted in Raman spectroscopy studies,^58^ implying an extremely weak phonon decay, for example, via phonon–phonon scattering, consistent with both the long-lived CP signal observed here and its negligible anharmonicity (constant frequency over 10 ps). Finally, by performing a Gaussian fitting procedure for the positive PB signal corresponding to the A′ and B′ contributions, as shown in Figure 2e, we can obtain the temporal dynamics of the peak energies (Figure 2f). Both PB peaks are initially red-shifted by the photoexcitation and follow similar recovery dynamics. We find that the peak energies of both A′ and B′ resonances are also modulated (with matching phase) by the *A*_1g_ vibration with an amplitude of ∼2.5 meV or 5 meV peak-to-peak, as highlighted in the inset of Figure 2f.

For the excitation mechanism of CPs, two main processes are customarily invoked: impulsive stimulated Raman scattering (ISRS),^59^ and displacive excitation of coherent phonons (DECP).^60^ In ISRS, the CPs have a pump energy dependence that follows the excitation profile of the Raman tensor. The pump pulse transmits kinetic energy to the lattice atoms during a time interval much shorter than the oscillation period. At *t* = 0, the atoms are in a quasi-equilibrium position, resulting in a sine oscillation. In DECP, on the other hand, the population of excited states changes the potential energy surface and thus the quasi-equilibrium position of the lattice. Therefore, at *t* = 0, the lattice is at a maximum or minimum of the oscillating nuclear coordinate, resulting in cosine oscillation. The high time resolution of our experiment allows a precise determination of the phase of the oscillatory Δ*R*/*R* component and the identification of the cosine oscillation characteristic of DECP (see Figure S4 in the Supporting Information). Moreover, we find that the oscillations in MoTe_2_ have similar magnitudes, or are slightly enhanced, for excitation at 2.36 eV, compared to 1.91 eV (see Figure S5 in the Supporting Information), while the Raman peak at 170 cm^−1^ is much weaker for excitation energy at 2.33 eV, corroborating the identification of different excitation mechanisms for Raman scattering and CPs.

The spectral window where CP modulation of Δ*R*/*R* is visible, as seen in Figures 3a and 3b, can be divided into two regions: above and below 1.89 eV, where the oscillations have opposite sign equivalent to a phase difference of π. The Fourier transform of the CP in Figures 3b and 3c shows a single prominent mode with a constant frequency of 5.15 THz over the entire probe window. Its amplitude, however, as shown in Figure 3f, changes dramatically over the range of 1.7–2.3 eV, with a maximum at or below the lowest probe energy (1.72 eV), and a zero at ∼1.89 eV associated with a π-phase flip occurring at this energy, which is directly visible in the data in Figure 3d, and confirmed by Fourier analysis in Figure 3e.

**Figure 3:**
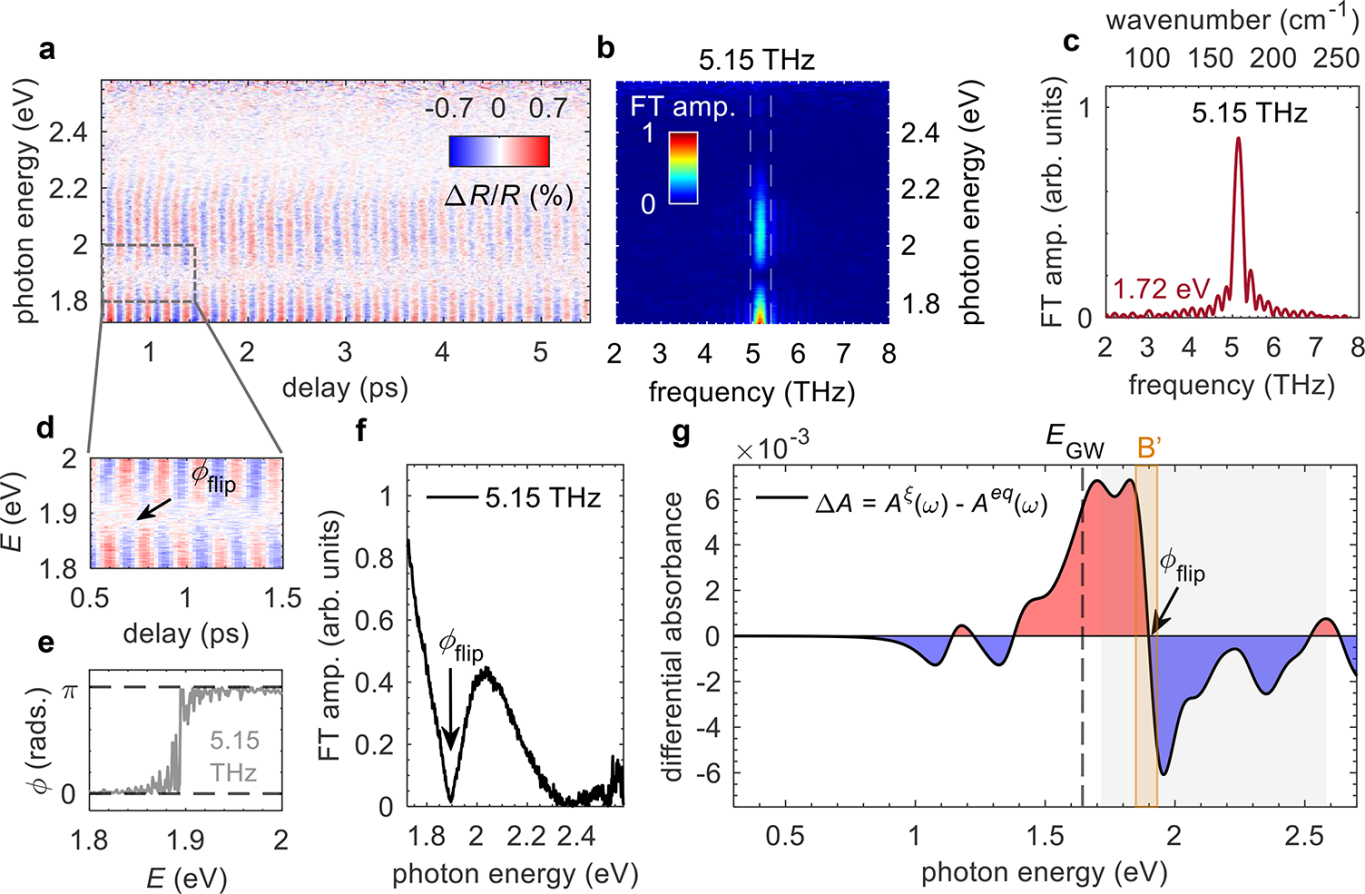
Frequency analysis of coherent phonon oscillations in monolayer 2*H*-MoTe_2_. (a) Coherent component of the transient signal map, Δ*R*/*R*. (b) Fourier transform (FT) map of the data in panel a. (c) FT frequency spectrum extracted at 1.72 eV, showing a peak at ∼5.15 THz (∼172 cm^−1^). (d) Coherent component map showing the energy range 1.8–2.0 eV near to the phase flip, labeled ϕ_flip_. (e) FT phase spectrum confirming the phase flip from 0 to π, which occurs at ∼1.89 eV. (f) FT amplitude spectrum extracted at 5.15 THz (vertical dashed area in panel (b)). (g) Differential absorbance spectrum, *A*^ξ^(ω) – *A*^eq^(ω) from ab initio calculations. The vertical dashed line indicates the GW direct bandgap energy, while the orange shaded area shows the B′ exciton transition. The gray shaded area highlights the experimentally explored energy range.

To gain further insight into the probe energy dependence of the amplitude and phase of the 5.15 THz vibration, we calculated the change in the absorption spectrum caused by out-of-plane displacement of the Te atoms around the central Mo along the *c*-axis, mimicking an *A*_1g_ -type oscillation launched by the pulse. Starting from the result of the BSE calculations, as described in the Methods section of the Supporting Information, we obtain the polarizability per unit area, α_2D_(ω), and define an effective dielectric tensor, ϵ_2D_(ω) which takes into account the effects of quantum confinement in two dimensions:^61^
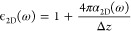
where Δ*z* is the material thickness. In the case of monolayer MoTe_2_, the theoretical value is Δ*z* = 7.66 Å. The dielectric tensor is used to compute the absorbance, according to the expression^62^
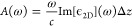
The differential absorbance, Δ*A* was then calculated by subtracting the absorbance of the equilibrium structure, *A*^eq^ from the absorbance, *A*^ξ^(α) with atoms displaced by a fixed amount, α along the *A*_1g_ phonon mode, ξ. The differential absorbance measured experimentally is small enough to be described in terms of a linear dependence on the atomic displacement α, i.e., *A*^ξ^(α) ≃ *A*^eq^ + ∂_α_*A*^ξ^α. In the numerical simulations, α was fixed to obtain a stretching along the *c*-axis equivalent to 0.5% of the Mo–Te bond length, measured from the center of the atoms.

The result, shown in Figure 3g, reproduces both the Fourier spectrum and the phase flip remarkably across the experimental energy window (gray-shaded area). The calculated spectrum reveals a rich structure consisting of multiple optical transitions, where the peak widths have been inferred. Most notably, the spectrum is dominated by a large contribution centered at ∼2 eV, which corresponds to the B′ exciton absorption, suggesting a particularly strong coupling of this transition with the *A*_1g_ mode. The theoretical phase flip energy was found to be 1.90 eV, in almost perfect agreement with that observed experimentally.

We now inspect the effects of the *A*_1g_ mode atomic displacement on the electronic and optical properties of MoTe_2_ in more detail. Figure 4a shows calculations of the optical absorption of a monolayer in equilibrium, including excitonic effects, compared to stretching (red line) and compression (blue line) of 0.5% along the *c*-axis. The main features (labeled in Figure 4a) are reproduced with excellent agreement to previous works^42,55^ starting from the lowest energy A exciton at ∼1.1 eV. In particular, we emphasize the position of the B′ transition at ∼2 eV, which is located within the continuum beyond the GW direct bandgap, and, hence, a large number of transitions (orange-shaded area) have been taken into account to analyze it correctly. The optical absorption is subtly different except for the spectral region of 1.6–2.1 eV, where there is dramatic rearrangement in both energy and magnitude. For out-of-plane compression, the A′ and B′ resonances shift to higher energies, with the B′ peak shifting more significantly, which leads to a larger separation of the two overlapping peaks, while upon stretching they shift to lower energies, increasing the overlap and forming a single, more intense peak. Consistently with our experimental results, the strongest modulation of the absorption occurs on the low energy side of the double peak with a relative change of ∼10% for 0.5% out-of-plane displacement.

**Figure 4:**
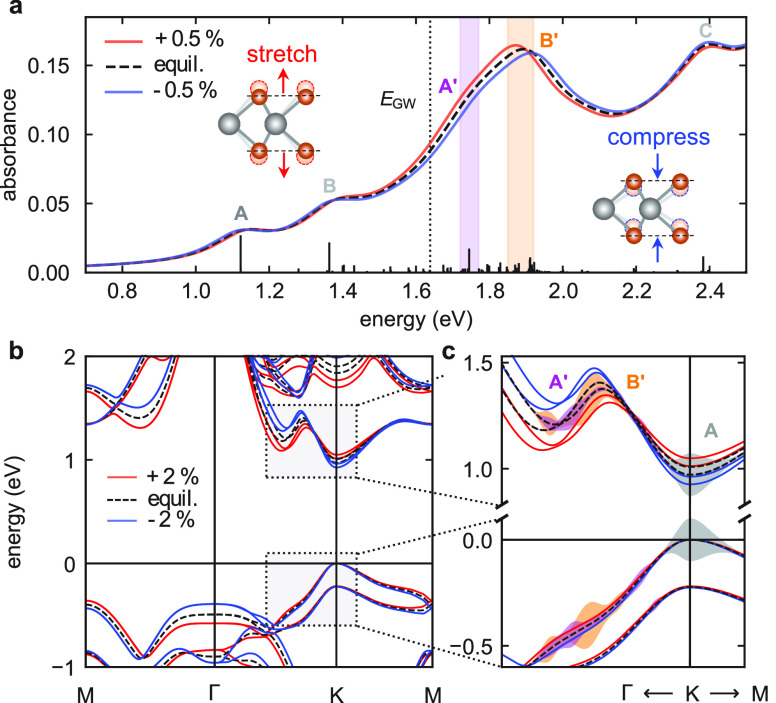
Optical and electronic properties of monolayer 2*H*-MoTe_2_ from ab initio calculations. (a) Optical absorption spectra for vertical stretching (red) and compression (blue) along the *c*-axis direction of the Mo–Te bond length equivalent to 0.5%. The spectrum of the equilibrium structure (dashed line), i.e., no stretching or compression, is shown for comparison. The purple- and orange-shaded areas indicate the A’ and B’ exciton resonances, respectively. The dashed vertical line is the GW direct bandgap energy, *E*_GW_. The black vertical bars correspond to the poles of the excitonic matrix, where their height is proportional to the oscillator strength. (b) Electronic band structure for 2% stretching (red) and compression (blue). (c) Selected region around the *K*-point. The shaded areas represent the energy-momentum distribution of the optical transitions related to the A, A′, and B′ excitons for the equilibrium structure, as indicated.

The particularly strong coupling of the B′ exciton with the *A*_1g_ mode leads to an energy shift of the absorption around the equilibrium position as the atoms oscillate. Such an energy modulation was observed in the experiments (Figure 2f), as discussed previously. In the displacive excitation of coherent phonons, photoexcitation changes the nuclear quasi-equilibrium positions, and at *t* = 0 the nuclei are displaced relative to this new quasi-equilibrium. Their oscillation results in a periodic modulation of the absorption spectrum. Following the oscillatory component at energies below 1.89 eV, we observe a positive Δ*R*/*R* at *t* = 0. Therefore, at *t* = 0 the spectrum is blue-shifted relative to its new quasi-equilibrium, meaning the out-of-plane positions of the Te atoms are at a minimum. Their new quasi-equilibrium position is at a larger distance than prior to photoexcitation. Above 1.89 eV the same blue shift results in an increase of the absorption and hence an oscillatory modulation of opposite sign, i.e., a phase shift of π.

To understand why the optical absorption exhibits such a dramatic change in a specific energy range, we now analyze the calculated electronic band structures, shown in Figures 4b and 4c, for a larger displacement of 2% in order to emphasize the effects. The calculations confirm the two valence band maxima and conduction band minima at the *K*-point, which give rise to the lowest energy excitonic transitions A and B, outside the spectral window of our experiment. The dominant change in the electronic structure at the *K*-point is bandgap renormalization, which results in a comparatively small energy shift of the A exciton transition. Instead, the A′ and B′ transitions, which appear as a broad double PB peak centered at ∼1.9 eV in the experiment, originate from regions of the band structure along the *K*–Γ direction, as illustrated by the shaded areas in Figure 4c. Here, we find that the atomic displacement results in considerable modification of the bands, especially close to the local minima and maxima of the conduction band, where optical transitions related to the A′ and B′ exciton are most important. The result is a large energy shift and change in the magnitude of the optical absorption in the spectral range close to these transitions, as shown in Figure 4a. We note that the A′ and B′ peaks in MoTe_2_ are much narrower in optical absorption, compared to high energy transitions present in other TMDs, such as the C peak in MoS_2_, where the oscillatory maximum is found.^28^ This may partially contribute to the stronger coherent response, as the transient signal is proportional to the first derivative of the absorption. In addition, by calculating the orbital character of the projected (PDOS) band structure, we find that the portion of the conduction band related to the A′ and B′ transitions is strongly hybridized with Te orbitals (up to ∼40%), while at the *K*-point, it is mostly Mo (see Figure S6 in the Supporting Information). Since the *A*_1g_ vibration involves the out-of-plane motion of Te atoms around the fixed Mo atom, the strongly hybridized regions of band structure are most sensitive to the change in the interatomic distance, i.e., variation in Mo–Te orbital proximity, resulting in a significant energy renormalization. Hence, this explains the extremely intense coherent oscillations observed in 2*H*-MoTe_2_ as the result of a modulation of the optical absorption due to the out-of-plane atomic displacement launched by the optical pulse.

In conclusion, we investigated the generation and detection of coherent phonons in monolayer 2*H*-MoTe_2_ using a combination of femtosecond pump–probe microscopy and ab initio calculations. In excellent agreement between experiment and theory, we found that photoexcitation stimulates the out-of-plane *A*_1g_ vibration, which strongly modulates the absorption in the visible range, especially around the A′ and B′ excitons. We identified a displacive excitation mechanism where photoexcitation shifts the quasi-equilibrium positions of the Te atoms to a larger out-of-plane distance. Our calculations also predict modulations of significant magnitude around the A and B excitons, thus expanding the potential for the coherent control of optical phonons and excitons via optical excitation or applied out-of-plane compressive strain into the NIR region down to ∼1 eV.

## Supporting Information Available

Details of experimental and computational methods, exciton decay dynamics, damping time including temperature dependence, oscillatory phase analysis, excitation energy dependence, projected band structure calculations (PDF)
