# Supplementary material for: Strong Coupling of Coherent Phonons to Excitons in Semiconducting Monolayer MoTe2
Source: Nano Lett. 2023 Sep 26;23(20):9235–42. doi: 10.1021/acs.nanolett.3c01936 (PMC10603802; doi:10.1021/acs.nanolett.3c01936)
Supplement: Supplementary file 1 — nl3c01936_si_001.pdf [file nl3c01936_si_001.pdf]

Supporting Information for: Strong Coupling of Coherent Phonons to Excitons in Semiconducting Monolayer $MoTe_2$.

Charles J. Sayers,[†] Armando Genco,[†] Chiara Trovatello,[†,‖] Stefano Dal Conte,[†] Vladislav O. Khaustov,[‡,⊥] Jorge Cervantes-Villanueva,[¶] Davide Sangalli,[§] Alejandro Molina-Sanchez,[¶] Camilla Coletti,[‡,#] Christoph Gadermaier,[†] and Giulio Cerullo[*,†]

†*Dipartimento di Fisica, Politecnico di Milano, 20133 Milano, Italy*

‡*Center for Nanotechnology Innovation @ NEST, Istituto Italiano di Tecnologia, 56127 Pisa, Italy*

¶*Institute of Materials Science (ICMUV), University of Valencia, Catedrático Beltrán 2, E-46980 Valencia, Spain*

§*Division of Ultrafast Processes in Materials (FLASHit), Istituto di Struttura della Materia-CNR (ISM-CNR), Area della Ricerca di Roma 1, 00016 Monterotondo, Scalo, Italy*

‖*Department of Mechanical Engineering, Columbia University, New York, New York 10027, United States*

⊥*Scuola Normale Superiore, Piazza San Silvestro 12, 56127 Pisa, Italy*

#*Graphene Labs, Istituto Italiano di Tecnologia, 16163 Genova, Italy*

E-mail: giulio.cerullo@polimi.it

Methods

Experimental

Ultrafast spectroscopy experiments were performed using a custom broadband transient absorption microscope setup based on a Ti:sapphire laser system (Coherent Libra) which outputs 100 fs pulses at 1.55 eV with 2 kHz repetition rate. The pump was generated using a non-collinear optical parametric amplifier (NOPA) tuned to a centre energy of $\sim$ 2.36 eV. The probe was created by white light continuum generation in a 1 mm sapphire plate using the laser fundamental. Pump and probe beams were focused onto the sample with a diameter of $\sim$ 5 and 3 µm , respectively, using an achromatic objective lens. Cross polarization was used to avoid scattering artefacts. The pump was pre-compressed using chirp mirrors to account for all transmissive optical elements in the beam path that introduce dispersion. The pulse compression was optimized by measuring a target metallic sample with a nearly instantaneous response, resulting in an overall temporal resolution of $\leq$ 40 fs, comparable to the build-up time of excitons in MoS_2 as measured previously.[1] A pump fluence of $\sim$ 500 $\mu J\,cm^{-2}$ was used. The sample was mounted inside an ultra low vibration closed-cycle cryostat, and the temperature maintained at $T = 10$ K. The setup operates in backscattering geometry, whereby the reflected probe is dispersed by a spectrometer onto a CCD camera. The differential reflectance of the sample ($\Delta R/R$) is then obtained by modulating the pump using an optical chopper, while the pump-probe delay is controlled by a mechanical delay line.

Computational

Theoretical investigation of the absorbance has been carried out by means of *ab initio* simulations. DFT[2,3] and DFPT[4] calculations were performed with *QUANTUM ESPRESSO*,[5–7] where the Perdew–Burke–Ernzerhof exchange-correlation functional[8] and a plane-wave cutoff at 50 Ha were used. To replace the effect of core electrons, fully relativistic pseudopoten-

tials were used from PSEUDODOJO[9] since spin-orbit coupling is taken into account. A vacuum distance of 40 a.u. was imposed in order to avoid the fictitious interactions between periodic layers. To calculate the quasiparticle correction, the GW method[10,11] was used in single-shot mode G_0W_0, applying the plasmon-pole approximation. In order to compute the optical properties taking into account the excitonic effects, the Bethe-Salpeter equation (BSE)[12] was solved, where the Tamm-Dancoff approximation was applied. 14 valence bands and 13 conduction bands were considered for the correct description of the optical absorption. The polarizability per unit area of the $MoTe_2$ monolayer was obtained from the BSE results, and used to calculate the effective dielectric function and the absorbance. In order to compute the coherent part of the differential absorbance, DFT and BSE simulations were first performed for equilibrium atomic positions and then with the atoms displaced along the Raman active A_{1g} phonon eigen-mode ξ, as explained in Ref.[13] Both GW and BSE simulations were performed using *YAMBO* code.[14] All calculations were performed with a $42 \times 42 \times 1$ Monkhorst-Pack grid[15] for the Brillouin zone.

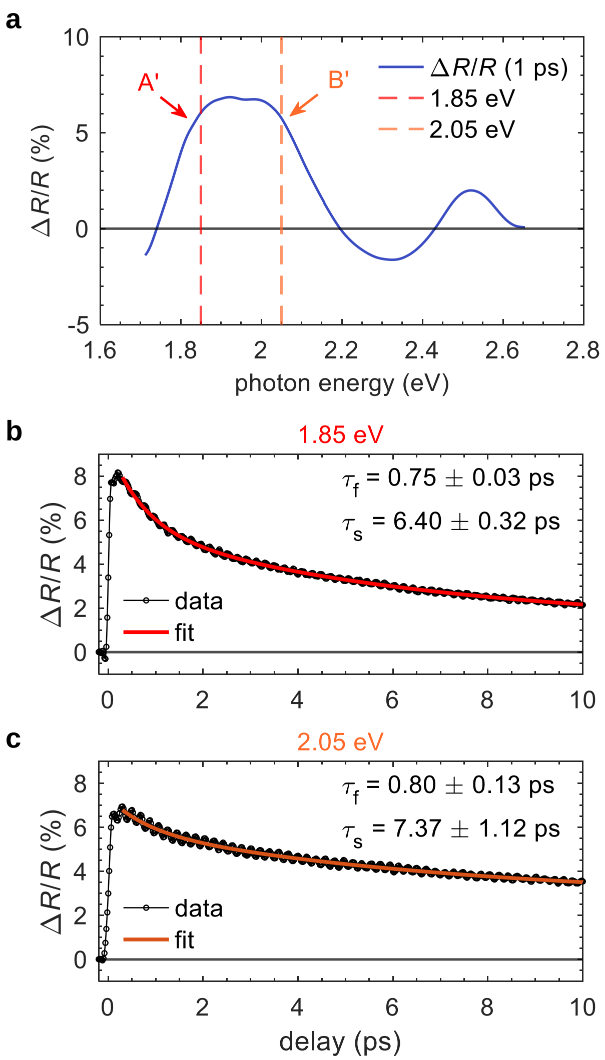

Figure S1: **Exciton decay dynamics obtained by multi-exponential fit.** (**a**) Transient $\Delta R/R$ spectrum at 1 ps. Vertical dashed lines indicate photon energies 1.85 and 2.05 eV selected for multi-exponential fitting which correspond to the approximate positions of the photobleaching (PB) signal of the A' and B' excitonic resonances. **(b) - (c)** Fit of the PB signal for each photon energy using a bi-exponential decay function $A_{\mathrm{f}}e^{-t/\tau_{\mathrm{f}}} + A_{\mathrm{s}}e^{-t/\tau_{\mathrm{s}}} + y_0$ that consists of a fast (τ_{f}) and slow (τ_{s}) component and a offset value, y_0. Data for $t > 0.3$ ps was used to fit only the decay dynamics. The results of the fitting, including associated errors, are reported in the figure panels.

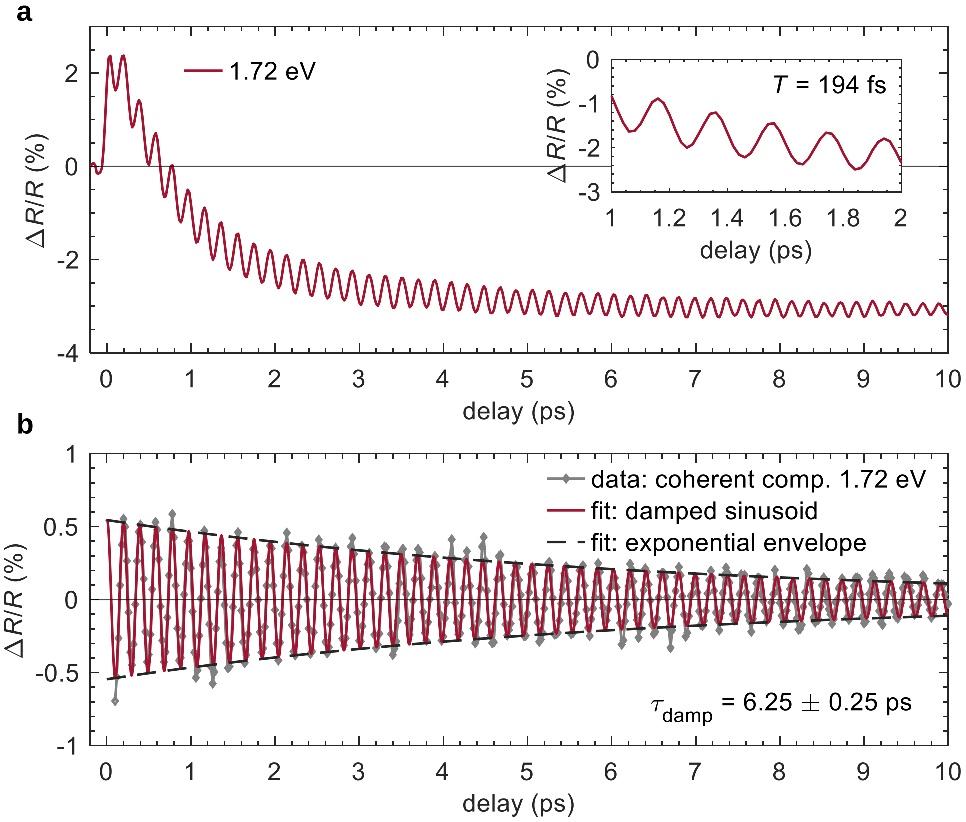

Figure S2: **Dephasing time obtained by damped sinusoidal fit.** (**a**) Dynamics selected at 1.72 eV near the maximum oscillatory signal, as seen in the main text. The inset shows a zoom of the data highlighting the $T = 194$ fs period (5.15 THz frequency). (**b**) Coherent component (grey dots) of the $\Delta R/R$ signal after subtraction of an exponential fit to the incoherent component. The coherent component data is fitted with a damped sinusoidal function $y(t) = Ae^{-t/\tau_{\mathrm{damp}}} \cos(\omega t + \phi)$ (solid red line) which gives a damping time of $\tau_{damp} = 6.25 \pm 0.25$ ps. The exponential envelope is shown for completeness (dashed lines).

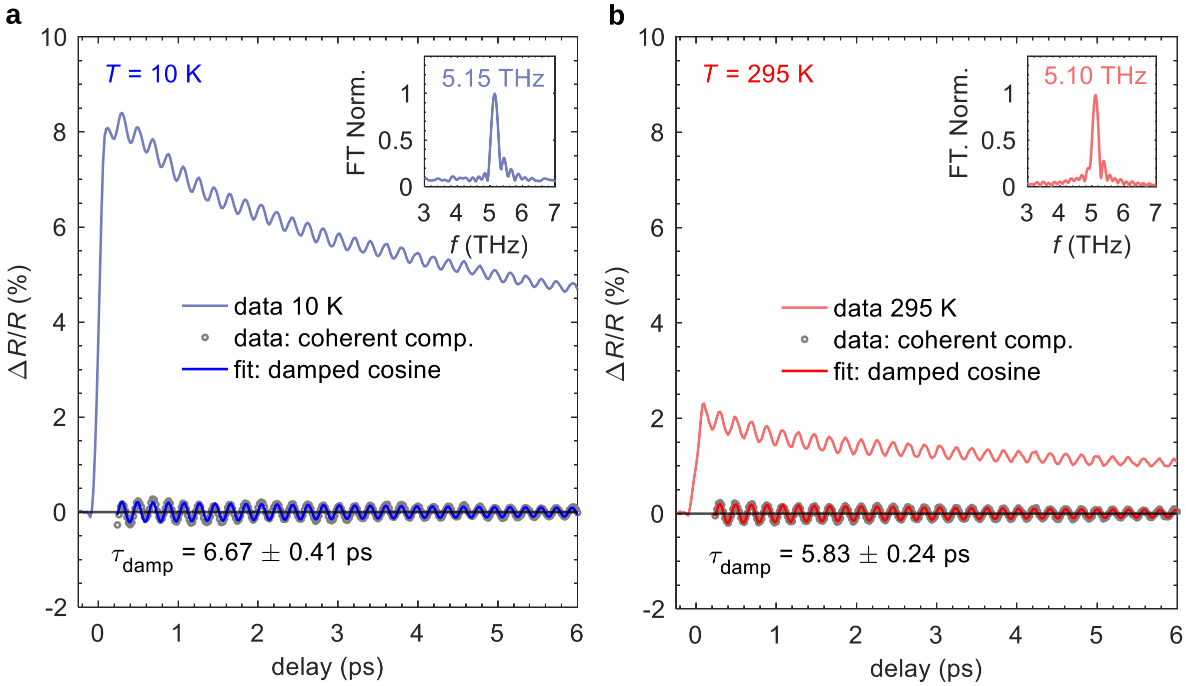

Figure S3: **Temperature-independent phonon lifetime** (**a**) 10 K dynamics selected at 2.00 eV near the average peak centre of the B' exciton. Following the same procedure as illustrated in Figure S2, the coherent component (grey dots) of the data is fitted with a damped sinusoidal function over the range (0.25 - 6.00) ps, which gives a damping time of $\tau_{damp} = 6.67 \pm 0.41$ ps. The inset shows the normalized Fourier transform of the coherent component with frequency $f = 5.15$ THz. (**b**) 295 K dynamics selected at 1.93 eV near the average peak centre of the B' exciton. The data was obtained from a different $MoTe_2$ sample than the one presented in the main text, and the pump fluence was $\sim 1000\ \mu\mathrm{J\,cm^{-2}}$. Following the same procedure as in panel (a), gives a damping time of $\tau_{damp} = 5.83 \pm 0.23$ ps. The inset shows the normalized Fourier transform of the coherent component with frequency $f = 5.10$ THz.

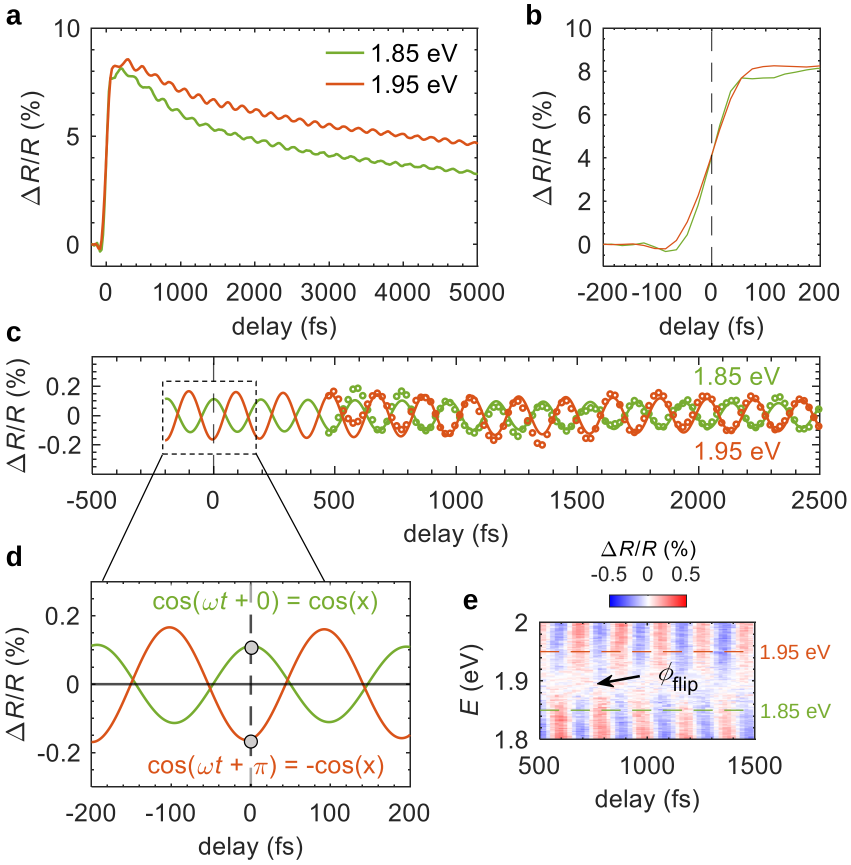

Figure S4: **Determination of the oscillatory phase.** (**a**) Dynamics at suitable probe photon energies of 1.85 eV and 1.95 eV are selected for the low and high energy side of the phase flip which occurs at $\sim$ 1.9 eV, as reported in the main text [see also panel (e)]. (**b**) The zero delay ($t = 0$) is confirmed by the half-rise time of the incoherent $\Delta R/R$ signal as expected for a Gaussian pulse width. (**c**) The coherent component dynamics at the selected photon energies (open circles) are fitted with a damped sinusoidal function (solid lines) in the range $+500$ to $+5000$ fs and then extrapolated to negative delay. (**d**) Zoom of the extrapolated fit showing a maximum or a minimum in the oscillatory signal at $t = 0$ for the 1.85 eV and 1.95 eV traces respectively, confirming the cosine nature of the coherent oscillations, and a π phase shift across $\sim$1.9 eV. (**e**) Portion of the coherent component map as reported in the main text close to the phase flip energy at $\sim$1.9 eV, highlighting the photon energies selected for the phase analysis.

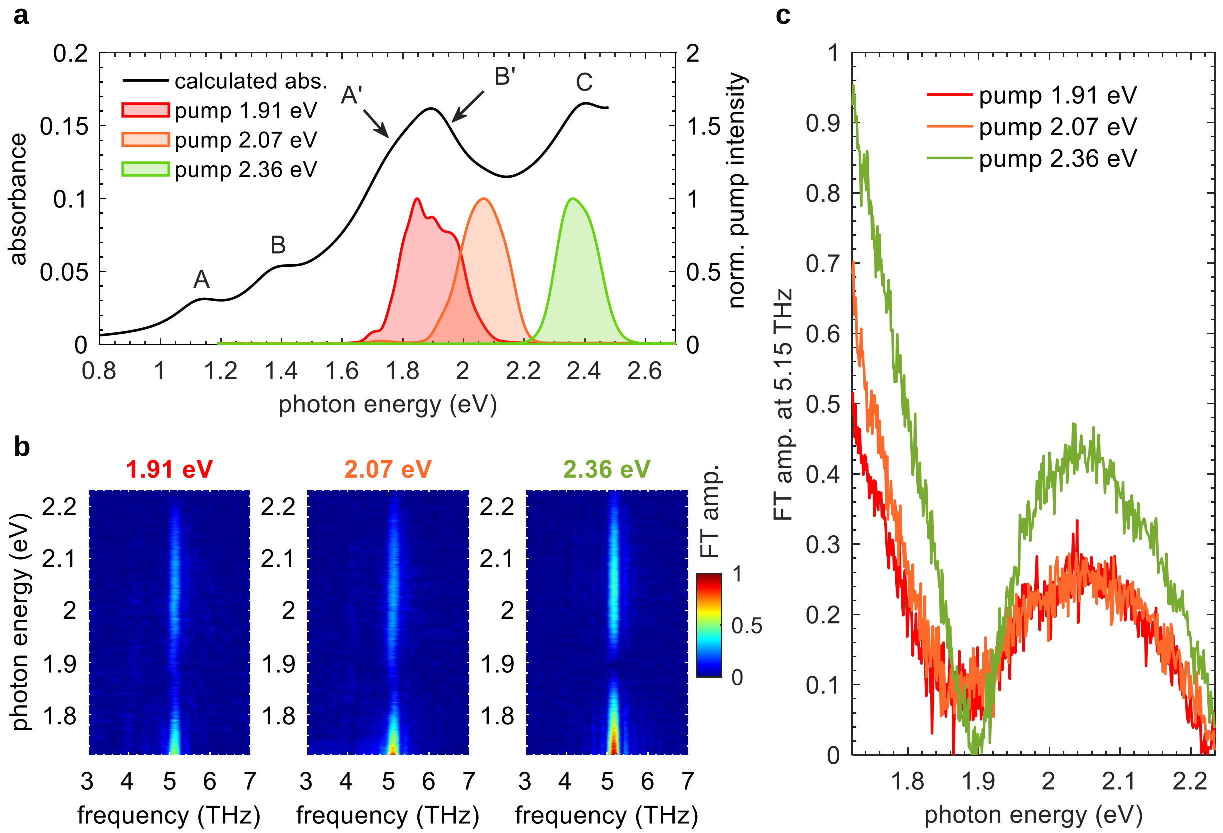

Figure S5: **Excitation energy dependence of the oscillation amplitude.** (**a**) Calculated optical absorbance (left axis) for the equilibrium structure of $2H$-$MoTe_2$. Normalized broadband pump spectra (right axis) with centre energies indicated in the legend. (**b**) Fourier transform (FT) map of the oscillatory signal component for each excitation (pump) energy. The pump fluence was $\sim 500\ \mu J\,cm^{-2}$ in all cases. (**c**) FT amplitude at 5.15 THz extracted from panel b for each excitation energy, revealing a similar spectral profile in all cases, and a slightly enhanced amplitude for higher pump energies.

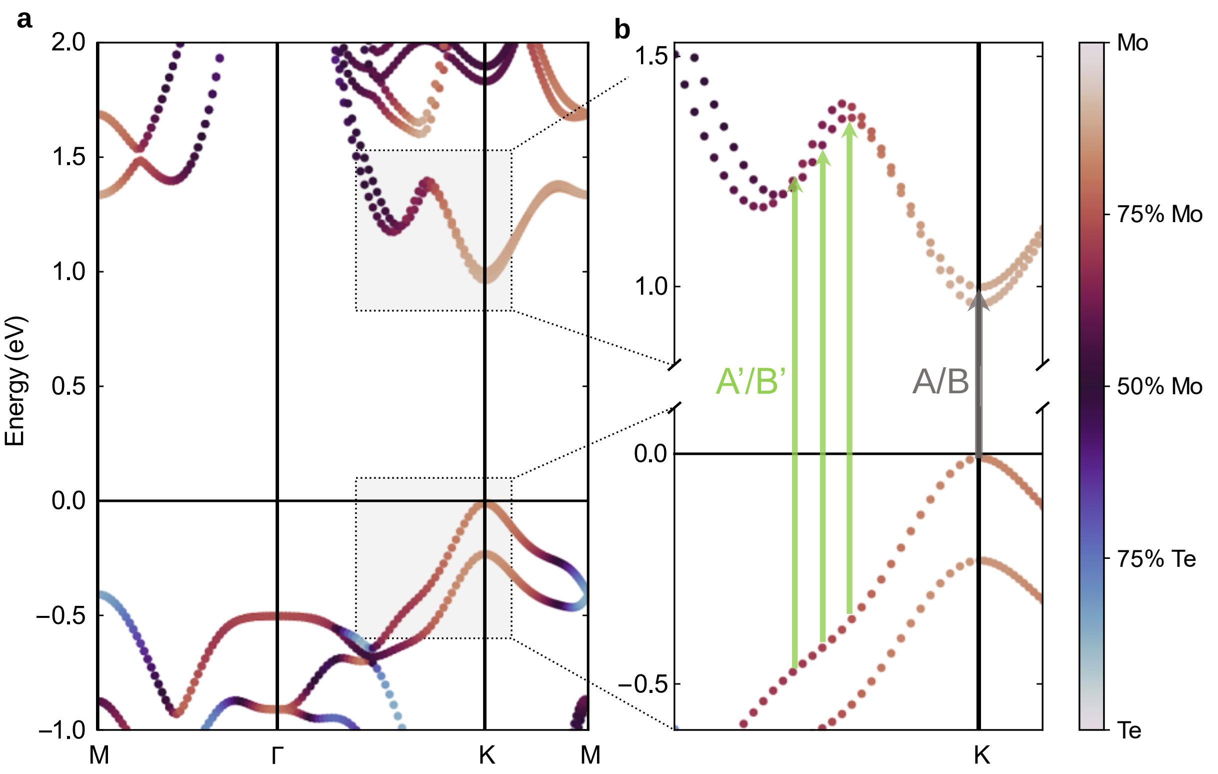

Figure S6: **Orbital character of the projected band structure from *ab initio* calculations.** (**a**) Electronic band structure for undistorted (equilibrium) monolayer 2H-$MoTe_2$, where the colour represents the projected density of states (PDOS) contributions from Mo and Te atomic orbitals expressed as a percentage. (**b**) Selected region around the K-point. The vertical arrows illustrate regions of the band structure related to the A/B excitonic transitions at K, and the A'/B' transitions along the $K-\Gamma$ direction.

References

(1) Trovatello, C.; Katsch, F.; Borys, N. J.; Selig, M.; Yao, K.; Borrego-Varillas, R.; Scotognella, F.; Kriegel, I.; Yan, A.; Zettl, A.; Schuck, P. J.; Knorr, A.; Cerullo, G.; Conte, S. D. The ultrafast onset of exciton formation in 2D semiconductors. *Nat. Comm.* **2020**, *11*, 5277, DOI: doi:10.1038/s41467-020-18835-5.

(2) Hohenberg, P.; Kohn, W. Inhomogeneous Electron Gas. *Phys. Rev.* **1964**, *136*, B864–B871, DOI: doi:10.1103/PhysRev.136.B864.

(3) Kohn, W.; Sham, L. J. Self-Consistent Equations Including Exchange and Correlation Effects. *Phys. Rev.* **1965**, *140*, A1133–A1138, DOI: doi:10.1103/PhysRev.140.A1133.

(4) Baroni, S.; de Gironcoli, S.; Dal Corso, A.; Giannozzi, P. Phonons and related crystal properties from density-functional perturbation theory. *Rev. Mod. Phys.* **2001**, *73*, 515–562, DOI: doi:10.1103/RevModPhys.73.515.

(5) Giannozzi, P. et al. QUANTUM ESPRESSO: a modular and open-source software project for quantum simulations of materials. *J. Phys: Condensed Matter* **2009**, *21*, 395502, DOI: doi:10.1088/0953-8984/21/39/395502.

(6) Giannozzi, P. et al. Advanced capabilities for materials modelling with Quantum ESPRESSO. *J. Phys.: Condensed Matter* **2017**, *29*, 465901, DOI: doi:10.1088/1361-648x/aa8f79.

(7) Giannozzi, P.; Baseggio, O.; Bonfà, P.; Brunato, D.; Car, R.; Carnimeo, I.; Cavazzoni, C.; de Gironcoli, S.; Delugas, P.; Ferrari Ruffino, F.; Ferretti, A.; Marzari, N.; Timrov, I.; Urru, A.; Baroni, S. Quantum ESPRESSO toward the exascale. *J. Chem. Phys.* **2020**, *152*, 154105, DOI: doi:10.1063/5.0005082.

(8) Perdew, J. P.; Burke, K.; Ernzerhof, M. Generalized Gradient Approximation Made Simple. *Phys. Rev. Lett.* **1996**, *77*, 3865–3868, DOI: doi:10.1103/PhysRevLett.77.3865.

(9) van Setten, M.; Giantomassi, M.; Bousquet, E.; Verstraete, M.; Hamann, D.; Gonze, X.; Rignanese, G.-M. The PseudoDojo: Training and grading a 85 element optimized norm-conserving pseudopotential table. *Comp. Phys. Comms.* **2018**, *226*, 39–54, DOI: doi:10.1016/j.cpc.2018.01.012.

(10) Onida, G.; Reining, L.; Rubio, A. Electronic excitations: density-functional versus many-body Green's-function approaches. *Rev. Mod. Phys.* **2002**, *74*, 601–659, DOI: doi:10.1103/RevModPhys.74.601.

(11) Reining, L. The GW approximation: content, successes and limitations. *WIREs Comput. Mol. Sci.* **2018**, *8*, e1344, DOI: doi:10.1002/wcms.1344.

(12) Marsili, M.; Molina-Sánchez, A.; Palummo, M.; Sangalli, D.; Marini, A. Spinorial formulation of the GW-BSE equations and spin properties of excitons in two-dimensional transition metal dichalcogenides. *Phys. Rev. B* **2021**, *103*, 155152, DOI: doi:10.1103/PhysRevB.103.155152.

(13) Miranda, H. P. C.; Reichardt, S.; Froehlicher, G.; Molina-Sánchez, A.; Berciaud, S.; Wirtz, L. Quantum Interference Effects in Resonant Raman Spectroscopy of Single- and Triple-Layer $MoTe_2$ from First-Principles. *Nano Lett.* **2017**, *17*, 2381–2388, DOI: doi:10.1021/acs.nanolett.6b05345.

(14) Sangalli, D. et al. Many-body perturbation theory calculations using the yambo code. *J. Phys: Condensed Matter* **2019**, *31*, 325902, DOI: doi:10.1088/1361-648X/ab15d0.

(15) Monkhorst, H. J.; Pack, J. D. Special points for Brillouin-zone integrations. *Phys. Rev. B* **1976**, *13*, 5188–5192, DOI: doi:10.1103/PhysRevB.13.5188.
